# Coagulation Profile in Neonates with Congenital Heart Disease: A Pilot Study

**DOI:** 10.3390/medicina60020268

**Published:** 2024-02-03

**Authors:** Paraskevi Papadogeorgou, Serena Valsami, Maria Boutsikou, Eleni Pergantou, Aimilia Mantzou, Ioannis Papassotiriou, Zoi Iliodromiti, Rozeta Sokou, Elena Bouza, Marianna Politou, Nicoletta Iacovidou, Theodora Boutsikou

**Affiliations:** 1 Neonatal Department, Aretaieio Hospital, Medical School, National and Kapodistrian University of Athens, 115 28 Athens, Greece; 2 Blood Transfusion Department, Aretaieio Hospital, Medical School, National and Kapodistrian University of Athens, 115 28 Athens, Greece; 3 Haemostasis Unit/Haemophilia Centre, “Aghia Sophia” Children’s Hospital, 115 27 Athens, Greece; 4 First Department of Paediatrics, Medical School, National and Kapodistrian University of Athens, “Aghia Sophia” Children’s Hospital, 115 27 Athens, Greeceipapassotiriou@gmail.com (I.P.); 5 2nd Neonatal Intensive Care Unit, “Aghia Sophia” Children’s Hospital, 115 27 Athens, Greece

**Keywords:** neonates, congenital heart disease, coagulation abnormalities, ADAMTS-13

## Abstract

*Background and Objectives*: congenital heart disease (CHD), cyanotic and, to a lesser degree, acyanotic, often are accompanied by coagulation abnormalities, impacting substantially morbidity and mortality. Until now, no consistent hemostatic patterns have been demonstrated in neonates and children with CHD because they represent a variable and heterogenous population. The aim of the present study is to investigate the hemostatic profile, as well as the role of ADAMTS-13 (a disintegrin and metalloprotease with thrombospondin type-1 motives), the cleaving protein of von Willebrand factor (VWF) in neonates with CHD and compare them to healthy age-matched controls. *Materials and Methods*: twenty neonates with a mean gestational age of 37.1 ± 2.5 weeks were included in the CHD group, and 18 healthy neonates with a mean gestational age of 38.2 ± 1.5 weeks were in the control group. *Results:* prothrombin time was significantly prolonged, and accordingly, factor VII (FVII) levels were significantly decreased in the CHD group in comparison to controls. Factor VIII (FVIII), VWF, and ristocetin cofactor activity (Rcof) levels were significantly higher in the study vs. control group. Concentrations of ADAMTS-13 were decreased in the CHD vs. control group, but the difference was not statistically significant. Our results, in combination, indicate a balanced hemostatic mechanism, although with greater variability in neonates with CHD, while developmental aspects of coagulation are evident in the specific patient population. *Conclusions:* the coagulation profile is moderately impaired early in the course of CHD, though increased thrombogenicity is already present and should not be ignored.

## 1. Introduction

Congenital heart disease (CHD) is the most common congenital anomaly, with an estimated prevalence of moderate to severe CHD, about 6 cases per 1000 births [1]. They are typically classified into cyanotic and acyanotic. The etiopathogenesis of the disease is not clear, but the hypothesis encompassing the interaction of genetic and environmental factors, first proposed by Nora in 1968, is still the most dominant [2]. Coagulation abnormalities in patients with CHD were first described more than 70 years ago. Bahnson and Ziegler first reported, in 1950, severe hemorrhage associated with significant mortality after a Blalock–Taussig shunt, and 2 years later, Hartman reported lethal bleeding in patients undergoing surgery for cyanotic CHD [3,4]. Despite advances in the management of CHD and associated coagulation abnormalities, approximately 5% of open-heart surgery in children is complicated by perioperative bleeding, while thrombosis is also a substantial problem, affecting children even a long time after recovery from surgery [5].

The aim of the present study is to investigate the hemostatic mechanism, as well as the implication of the ADAMTS-13 protein (a disintegrin and metalloprotease with thrombospondin type-1 motives protein) in the coagulation process in neonates with CHD and compare them to healthy age-matched controls. ADAMTS-13 is the cleaving protein of von Willebrand factor (VWF), which is released into the circulation in the form of ultra-large multimers (UL-VWF). UL-VWF exert high affinity for platelets, promoting platelet aggregation and microthrombi formation. Thus, the ADAMTS-13’s role is crucial to control their size and associated enhanced thrombogenicity [6]. ADAMTS-13 deficiency is associated with thrombotic thrombocytopenic purpura (TTP), although there is growing evidence that it is implicated in many other conditions, such as ischemic stroke, myocardial infarction, sepsis, and perioperative thrombotic complications in neonates and infants [7]. Investigation of ADAMTS-13 implication in CHD is of great significance since ADAMTS-13 levels may have a great impact on hemorrhagic and thrombotic complications in CHD patients, especially neonates. Nonetheless, recombinant ADAMTS-13 substitution may prospectively serve as a promising therapeutic intervention in patients with CHD, as ADAMTS-13 replacement therapy is currently under investigation for hereditary TTP and several other conditions, such as infection, myocardial infarction and thrombotic microangiopathy [8,9].

## 2. Materials and Methods

This is a prospective study including term and preterm neonates with CHD and healthy neonates who served as controls. Neonates with CHD were hospitalized at the 2nd Neonatal Intensive Care Unit of Aghia Sophia Children’s Hospital in Athens, while controls were born at Aretaieio Hospital, National and Kapodistrian University of Athens. The study period extended from July 2017 to July 2020. The study was approved by the Hospitals’ Ethics Committees, and informed consent was signed by parents prior to recruitment. Twenty newborns with CHD and 18 healthy age-matched neonates were the study subjects.

The hemostatic profile in the CHD group was tested at baseline during a stable state of disease prior to surgery or cardiac catheterization, which could impair the coagulation parameters. In a small subgroup of 6 neonates, hemostatic status was also assessed after complete or partial surgical correction or heart catheterization in an attempt to investigate any alterations of clotting and anticlotting factors before and after the invasive procedure. Exclusion criteria for the control group were maternal thrombophilia or any other maternal coagulation disorder, maternal intake of anticoagulation treatment, aspirin and antiepileptic medication, diabetes (gestational, type 1 or 2), preeclampsia, maternal lupus erythematosus, perinatal stress (defined as Apgar score 5′ < 7), and intrauterine growth restriction, cardiorespiratory morbidity, infection, or congenital anomalies in the neonate.

Blood was collected in 3.5 mL 9NC coagulation sodium citrate 3.2% containing VACUETTE^®^ TUBE, Greiner Bio-One GmbH, Kremsmünster, Austria. All samples were assessed at the Haemostasis Unit/Haemophilia Centre, Aghia Sophia Children’s Hospital. The following parameters were tested: prothrombin time (PT), activated partial thromboplastin time (APTT), fibrinogen, factors II (FII), VII (FVII), VIII (FVIII), IX (FIX), X (FX), and VWF, ristocetin cofactor activity (Rcof), antithrombin (AT), proteins C and S (PC and PS, respectively), D-dimers and ADAMTS-13 protein. PT, APTT, Fibrinogen, FII, FVII, FVIII, FIX, and FX were tested at Instrumentation Laboratory ACL TOP 700, Werfen, Bedford, Massachusetts, USA and the reagents used were HemosIL, Instrumentation Laboratory Company, Werfen, Bedford, MA, USA. VWF, Rcof and D-dimers were tested at BCS®XP System, SIEMENS Healthineers, Forchheim, Germany and the corresponding reagents used were VWF Ag, INNOVANCE® VWF Ac, INNOVANCE D-Dimer, SIEMENS Healthineers, Marburg, Germany. AT, PC and PS were measured using chromogenic method at BCS®XP System, SIEMENS Healthineers, Forchheim, Germany and the reagents used were Berichrom® Antithrombin A, Berichrom Protein C and Protein S Ac, SIEMENS Healthineers, Marburg, Germany. ADAMTS-13 was measured using Human ADAMTS 13 Quantikine ELISA, R&D Systems, Minneapolis, Minesota, USA. 

## 3. Statistical Analysis

Statistical analysis was performed using SPSS (version 23; SPSS Inc., Chicago, IL, USA). One-way ANOVA was applied in order to detect differences in PT, APTT, Fibrinogen, FII, FVII, FVIII, FIX, FX, VWF, Rcof, AT, PC, PS, D-Dimers, and ADAMTS-13 among the study groups. Student’s T-test was used to examine differences in continuous variables among groups, while paired T-test was applied to examine differences in coagulation parameters between the preoperative and postoperative states. X2 chi-square test was applied to detect differences between categorical variables. Pearson’s correlation coefficient was used to examine possible correlations between continuous variables. *p* < 0.05 was considered statistically significant.

## 4. Results

**Figure 1:**
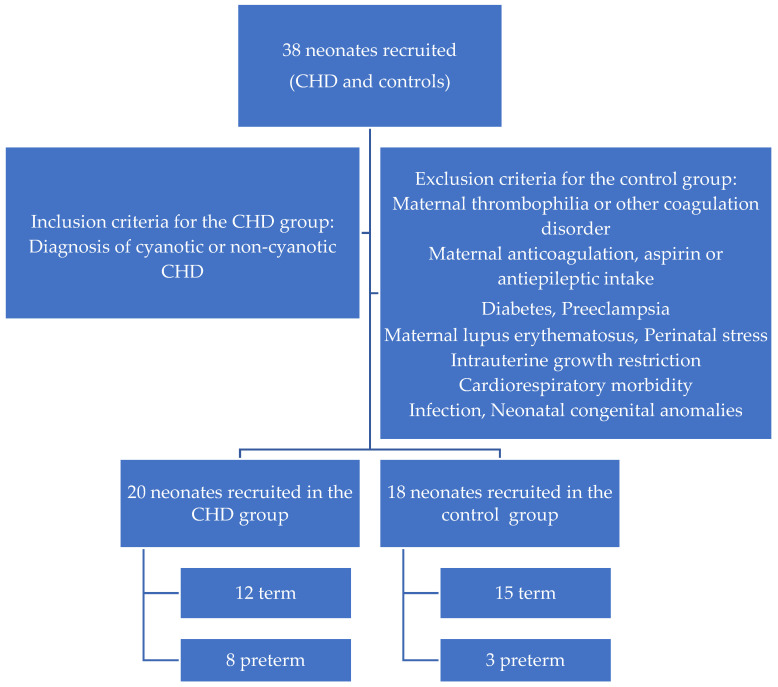
Demographic characteristics of the study population.

Comparisons of clotting, anticlotting factors, and ADAMTS-13 levels were undertaken between the CHD and the control group. The demographic characteristics of the two groups are depicted in Table 1. The mean chronological age at the time of assessment was 13.8 ± 14.0 days and 4.6 ± 2.2 days for the CHD and the control group, respectively. CHD was diagnosed antenatally in 45% of cases with routine obstetric follow-up. Demographic data and perinatal parameters are presented in Table 1 and Figure 1. Patient diagnoses are summarized in Table 2.

**Figure 2:**
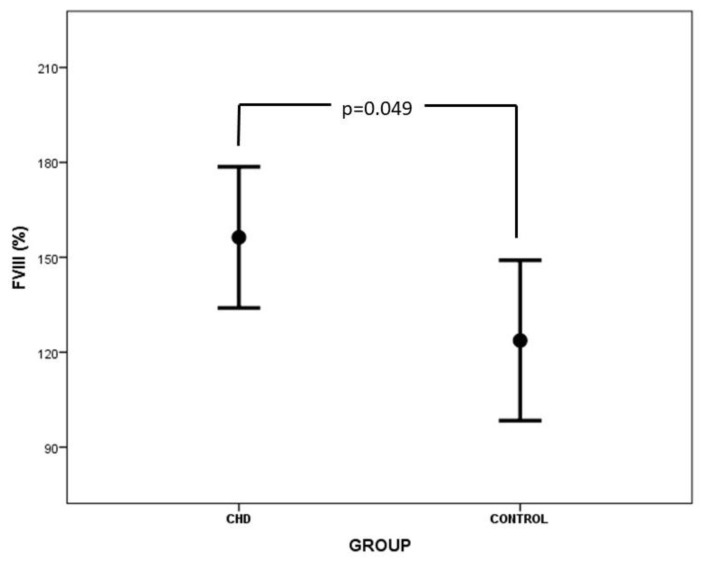
Comparison of FVIII levels between CHD and control group.

**Figure 3:**
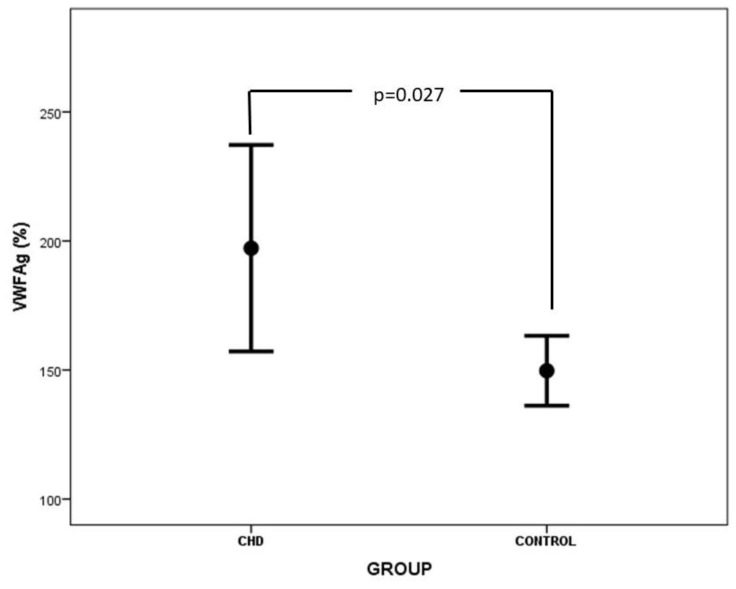
Comparison of VWF levels between CHD and control group.

The values of clotting and anticlotting factors, as well as ADAMTS-13 in neonates with CHD and controls, are presented in Table 3. Prothrombin time was significantly prolonged in neonates with CHD vs. controls (14.4 ± 3.8 s vs. 11.9 ± 1.8 s, *p* = 0.012). Similarly, FVII levels were significantly decreased in the CHD group vs. the control group (41.6 ± 22.4% vs. 60.1 ± 16.0%, *p* = 0.006). Concentrations of FVIII, VWF, and Rcof were statistically higher in neonates with CHD vs. controls (Table 3, Figure 2 and Figure 3). A comparison of ADAMTS-13 levels between the CHD and the control group did not reveal any significant difference, although lower ADAMTS-13 levels were reported in the CHD group. No considerable association was observed between VWF levels and the ABO blood group. The anticlotting mechanism was not impaired in neonates with CHD vs. controls. Interestingly, D-dimers were lower in the CHD group than in the control group (*p* = 0.036). Three out of 20 neonates with CHD demonstrated minor bleeding tendency, and two patients suffered clinically apparent thrombotic complications (inferior vena cava and right femoral vein thrombosis in the first case and Blalock–Taussing shunt occlusion in the second case).

Furthermore, coagulation parameters in the CHD group were analyzed in relation to the severity of the underlying heart defect, as assessed by the Bethesda classification system, and no significant difference was observed. However, PT was higher, and accordingly, FVII levels were lower in cases with adverse outcomes (12.4 ± 1.8 s vs. 16.8 ± 1.3 s, *p* = 0.001 and 54.6 ± 19.6% vs. 20.8 ± 3.5%, *p* = 0.005, respectively), indicating a clear association between coagulation profile and outcome. Impaired liver function, as defined by elevated direct or indirect bilirubin, increased hepatic enzymes, and decreased albumin concentration, was observed in 15.8% of patients. FVII levels were diminished in patients with impaired liver function (60.6 ± 12.6% vs. 143.7 ± 8.1%, *p* = 0.044), but no other coagulation factors were impaired.

Regarding gestational age, VWF levels were significantly higher and FII levels significantly lower in preterm newborns with CHD (163.8 ± 80.3% vs. 247.3 ± 70.3%, *p* = 0.028 and 67.3 ± 15.0% vs. 48.6 ± 12.0%, *p* = 0.014, respectively). Furthermore, AT concentrations were significantly increased in term neonates (74.2 ± 18.8% vs. 51.3 ± 7.3%, *p* = 0.004). Chronological age was positively correlated with levels of ADAMTS-13 (r = 0.549, *p* = 0.012), FII (r = 0.735, *p* = 0.001), FVII (r = 0.567, *p* = 0.009), AT (r = 0.654, *p* = 0.002) and PC (r = 0.530, *p* = 0.016). Birthweight was not associated with an impact on the hemostatic mechanism, as no difference was observed between small for gestational age (SGA, <10th percentile) and appropriate for gestational age (AGA, ≥10th percentile) neonates.

Fourteen neonates (70%) in the CHD group were prostaglandin-dependent, while 6 (30%) were not. A comparison of coagulation parameters between the two groups revealed that FVII, FX, PC, and PS levels are significantly higher in cases of CHD not dependent on prostaglandin infusion (Table 4). No statistically significant differences were observed in ADAMTS-13 and D-dimer levels between non-prostaglandin-dependent and prostaglandin-dependent groups (Table 4). Furthermore, FVII levels were significantly elevated in cases of CHD not requiring inotropes (68.0 ± 15.0% vs. 35.9 ± 20.6%, *p* = 0.021). No difference in the coagulation profile was observed in cases of CHD with an underlying genetic disorder.

## 5. Discussion

Our study represents a spherical assessment of the hemostatic mechanism in neonates with CHD and evaluates both clotting and anticlotting parameters during the early disease state. Growing evidence highlights the crucial role of ADAMTS-13 in the coagulation process. However, to the best of our knowledge, this is the first study comparing ADAMTS-13 levels between neonates with CHD and healthy age-matched controls.

Our study outlines that the coagulation profile is moderately impaired in neonates with CHD. PT was significantly prolonged in these neonates, probably due to lower levels of FVII. Prolongation of PT in cyanotic CHD has been reported since 1952. Many subsequent studies demonstrated the prolongation of clotting times in children with CHD. However, these studies demonstrated a more global impairment of the hemostatic profile, including decreased levels of FII, FV, FVIII, FIX, FX, and natural inhibitors of coagulation [4,10,11,12,13,14,15,16,17].

An increased bleeding tendency is substantial in children with CHD due to quantitative and qualitative platelet abnormalities, reduced levels of multiple coagulation factors, increased vascularity, and dilutional coagulopathy as a result of cardiopulmonary bypass implementation [18,19,20,21]. The prolongation of PT in association with reduced FVII levels observed in our study indicates that a derangement towards hemorrhage is evident early in the course of the disease, even preoperatively. Shebl et al. reported an increased liability both to hemorrhage and thrombosis in children with CHD, as demonstrated by thrombocytopenia, prolongation of clotting times, and increased levels of soluble markers of platelet and endothelial activation [17].

The underlying etiology of coagulation abnormalities is multifactorial, encompassing hypoxia, low cardiac output, venous congestion, consumption coagulopathy, liver and endothelial dysfunction, inflammation, platelet activation, defective vitamin-K carboxylation, secondary erythrocytosis, protein-losing enteropathy, and genetic predisposition [5,18,22,23,24,25]. Secondary erythrocytosis is a well-known compensatory mechanism for long-standing hypoxia, leading to hyperviscosity, vascular stasis, further deposition of platelets, coagulation activation, and finally, consumption coagulopathy [18,26]. Furthermore, hypoxia was associated with a prothrombotic state due to enhanced expression of clotting factors [23,27]. Heart failure and associated cardiac dilatation, systemic hypoperfusion, and blood stasis lead to increased prothrombotic tendency [16]. Odegard et al. demonstrated a clear association between ventricular dysfunction and multiple coagulation abnormalities [13]. In our study, major hemostatic alterations were not observed, probably due to the young age of the study population and the assessment of coagulation profile early in the clinical course of CHD. Furthermore, our study population represents a group of hospitalized patients under adequate medical support, leading probably to the elimination of coagulopathy’s pathophysiologic mechanisms. For example, ventricular dysfunction was counterbalanced by appropriate inotrope administration. Many of the aforementioned studies did not include an age-matched control group, and the comparisons were performed to levels published in the literature or adult reference values. In agreement with our study, Komp and Sparrow demonstrated moderate impairment of coagulation profile in children younger than 3 years old with cyanotic CHD prior to the development of polycythemia, while consumption coagulopathy developed only in older children after the onset of polycythemia [28].

Our study demonstrated higher levels of FVIII (Figure 2), VWF (Figure 3), and Rcof in neonates with CHD vs. controls. FVIII is synthesized by the liver sinusoidal cells, the endothelial and hematopoietic cells, and is expressed in many tissues like the liver, kidney, spleen, and lungs [29]. Liver dysfunction and associated inflammation of liver sinusoidal endothelium may up-regulate FVIII concentrations in plasma, although synthesis of other coagulation factors may be suppressed [30,31]. Moreover, endothelial dysfunction is common in CHD due to altered hemodynamics, the presence of central lines and infusate administration, and high shear stress due to hyperviscosity. Moreover, hypoxia augments the activation of neutrophils, leading to vasoactive and chemotactic substance release and further endothelial injury [32]. Levels of FVIII were associated with endothelial damage proportionally to its severity [33].

Endothelial perturbation is also associated with elevated levels of VWF, the carrier protein of FVIII, which stabilizes and protects FVIII from premature proteolytic degradation. VWF is synthesized by and stored mainly in endothelial cells and secondarily in megakaryocytes. Loss of endothelial integrity results in the release of VWF, a clear indicator of endothelial dysfunction. Furthermore, enhanced activation of platelets observed in children with cyanotic and acyanotic CHD may contribute to the increased levels of VWF [26,34,35]. Additionally, VWF and FVIII levels are highly interrelated, and increased endothelial release of VWF may lead to elevated FVIII levels in plasma [36]. Nevertheless, increased levels of FVIII and VWF may be attributed to their action as acute phase reactants, although other acute phase proteins, such as fibrinogen, were not found elevated in our study [37,38]. In line with our results, particular studies demonstrated normal or increased FVIII activity and antigen levels in patients with cyanotic CHD [11,28,39], while others yielded conflicting results [10,12,13,14,15]. Odegard et al. demonstrated increased levels of FVIII only after the Fontan procedure, a finding verified by other studies indicating an increased prothrombotic risk associated with Fontan physiology [30,40,41,42]. Binotto et al. also reported increased levels of VWF in children and adolescents with functionally univentricular physiology before completion of the Fontan operation, attributed to endothelial dysfunction [43]. Higher levels of VWF were also reported in cyanotic patients with Eisenmenger syndrome and in adult patients with previous myocardial infarction [34,44].

ADAMTS-13 levels were lower in neonates with CHD vs. controls but not statistically significant (*p* = 0.073). In contrast to our results, Wang et al. demonstrated significantly higher levels of ADAMTS-13 but lower ADAMTS-13 activity preoperatively in children with ventricular septal defect (VSD) [45]. Soares et al. reported lower levels of ADAMTS-13 antigen and activity in children with cyanotic CHD prior to surgical correction vs. controls. However, neonates were not included in the study [46]. Diminished ADAMTS-13 levels are probably attributed to elevated VWF levels, leading to consumption of ADAMTS-13, while liver dysfunction is an adding factor. ADAMTS-13 represents a negative acute phase protein, as demonstrated by our group and other investigators in previous studies [47,48]. Finally, hemostatic proteases, such as thrombin and plasmin, may proteolytically inactivate ADAMTS-13 [49]. To the best of our knowledge, this is the first study comparing ADAMTS-13 levels between neonates with CHD and healthy age-matched controls.

Interestingly, the anti-clotting mechanism was not impaired in our patient group, probably due to early assessment of the coagulation profile. Henriksson et al. also reported no impairment of AT levels in children with cyanotic CHD [11]. Conversely, other studies showed diminished levels of natural anticoagulants in children with CHD [10,12,13,14,15,30].

Several studies demonstrated that a substantial proportion of infants and children with thromboembolic disease have an underlying cardiac defect. Moreover, thromboembolic complications in children with CHD can be life-threatening and include venous, arterial, intracardiac, pulmonary, and central nervous system thromboembolism [50,51]. A hypercoagulable state was demonstrated in young cyanotic patients even prior to the onset of polycythemia, in accordance with the finding of pulmonary thrombi in infants with cyanotic CHD who decease before polycythemia develops [28]. Furthermore, a large retrospective study in patients less than 18 years old with CHD demonstrated an increasing rate of thrombosis after cardiac surgery, and the highest prevalence was observed in the neonatal population [52]. Nonetheless, Fontan physiology is clearly associated with an increased risk for thromboembolic events, even many years later [53]. Enhanced thrombogenicity in children with CHD was verified by studies that reported increased soluble platelet markers and endothelial activation [17,25]. Decreased levels of ADAMTS-13 and increased VWF:ADAMTS-13 ratio, as well as higher FVIII levels in our patient group vs. controls, indicate a significant prothrombotic risk factor in neonates with CHD. Elevated levels of FVIII in adults with deep vein thrombosis or pulmonary embolism were extensively described, while high plasma FVIII levels were linked to thrombosis in a dose-dependent manner [36,54,55,56,57]. With regards to children, elevated FVIII levels were reported in children with venous thrombosis and represent a poor prognostic factor associated with increased risk for recurrent thrombosis and post-thrombotic syndrome [58,59]. FVIII-associated thrombogenicity is attributed to its crucial role in the coagulation cascade and thrombin generation, an assumption verified by many in vivo and in vitro experiments, while elevated FVIII levels were associated with activated protein C resistance [36,60]. Increased preoperative VWF activity was linked to postoperative thrombosis in neonates and infants with CHD subjected to palliative or totally corrective cardiac surgery [61]. Nonetheless, VWF exerts a dose-dependent relationship with venous thromboembolic episodes in the adult population, though the underlying pathophysiologic mechanism is not clear [54,55,62]. Deficiency of ADAMTS-13 in our study, though not statistically significant, and the subsequent imbalance in the VWF:ADAMTS-13 axis represents a clear prothrombotic factor. Besides TTP, an imbalance in the VWF:ADAMTS-13 axis was linked to ischemic stroke and coronary occlusion and to increased risk for arterial and deep-vein thrombosis [63,64,65,66,67,68]. Katneni et al. demonstrated lower pre-operative levels of ADAMTS-13 antigen and activity in neonates with CHD who developed post-surgical thrombosis [7].

Impaired fibrinolysis, as well as loss of endothelial integrity and its anticoagulant properties, are additional prothrombotic risk factors in association with acquired risk factors, such as central line placement and infection [10,69,70].

The combined results of our study indicate that the coagulation profile is deranged towards both hemorrhage and thrombosis. Thus, the hemostatic equilibrium may be preserved in the particular patient population. However, taking into consideration both the laboratory data and clinical manifestations, we tend to conclude that a prothrombotic tendency is more dominant in our patient group. Nonetheless, conventional coagulation assays are not designed to evaluate the global hemostatic mechanism [71]. Future studies on newer viscoelastic techniques in neonates with CHD at an initial stable disease state would be of interest and give a thorough insight into the hemostatic status of these patients. In our study population, two subjects suffered clinically apparent thromboembolic complications of substantial severity. However, the true thrombotic incidence cannot be assessed, as thorough investigations of the vasculature, including angiography, were not generally undertaken. Nonetheless, low D-dimer levels exclude the diagnosis of thrombosis with some certainty. On the other hand, elevated D-dimer concentrations postoperatively in the small subgroup of 6 patients tested may be attributed to thrombotic complications besides surgery.

The identification of lower D-dimer levels in neonates with CHD vs. controls further contributes to the pre-existing controversy about the presence of disseminated intravascular coagulation (DIC) in patients with CHD, though the different chronological ages at the time of assessment could explain in part the discrepancy of results between the groups. First of all, D-dimer levels are 6–8 fold higher in healthy neonates in comparison to adults and remain elevated throughout the first year of life [72,73]. DIC of low-grade was reported in patients with CHD, and pulmonary microvasculature is thought to be the main area of localized thrombi formation [18,28,74]. However, in line with our study, other studies yielded conflicting results, putting in doubt the presence of DIC in patients with CHD [16,39,75,76,77]. Fibrinolysis is suppressed in CHD, in part due to the upregulation of plasminogen activator inhibitor-1 (PAI-1) induced by hypoxia [22,69]. The identification of endothelial dysfunction markers, such as FVIII and VWF, with normal or even low D-dimer levels indicates that endothelial damage precedes the activation of coagulation and fibrinolysis, as well as the development of intravascular thrombosis in neonates with CHD, in accordance with other studies [43].

Moreover, remarkable differences were demonstrated between prostaglandin-dependent and non-prostaglandin-dependent CHD. An enhanced tendency towards hemorrhage was shown in prostaglandin-dependent CHD vs. non-prostaglandin CHD, as verified by prolongation of PT and lower FVII and FX levels. On the other hand, thrombogenicity also seems to be greater in the prostaglandin-dependent group due to lower PC, PS, and ADAMTS-13 concentrations and higher D-dimers levels, although the differences in levels of ADAMTS-13 and D-dimers were not statistically significant. A more pronounced impairment of the hemostatic mechanism in prostaglandin-dependent CHD was not surprising, as these heart defects are more complex, and particular pathophysiologic mechanisms linked to coagulation abnormalities, such as secondary erythrocytosis and hypoxia are present only or mainly in cyanotic CHD. However, our results indicate that a delicate balance may be preserved in this patient population.

The results of our study suggest that developmental aspects of coagulation are still present in neonates with CHD in a similar pattern to healthy subjects. Chronological age was positively correlated with levels of ADAMTS-13, FII, FVII, AT, and PC in the CHD group, indicating that an evolutionary hemostatic status is present in these neonates. Odegard et al. also demonstrated a maturation pattern of coagulation profile in children with CHD, although at a slower rate than in healthy children [10,30].

Our study has some limitations. The sample size is small. However, it is well-defined, and an age-matched control group has been employed. A prospective longitudinal evaluation of coagulation parameters would elucidate the evolution of hemostatic abnormalities over time and in different disease states. However, this was out of the scope of the present study.

## 6. Conclusions

In conclusion, our study addresses an answer to the long-standing question of whether children with CHD per se have procoagulant and anticoagulant abnormalities. The hemostatic profile is deranged early in the course of CHD, although to a moderate degree. A delicate balance is probably retained, although it can be easily interrupted either by bleeding, thrombosis, or both. However, increased thrombogenicity is present in the particular patient population, and the isolated laboratory results favoring hemorrhage should not preclude the application of proper anticoagulation treatment. To the best of our knowledge, this is the first study undertaking proper comparisons of ADAMTS-13 implications in coagulation profile between neonates with CHD and healthy newborns. The role of recombinant ADAMTS-13 substitution in neonates, infants, and children with CHD should be explored in future studies since it represents a new and promising therapeutic tool.

## Figures and Tables

**Table 1:** Demographic data of the study participants.

Variable	CHD Group	Control Group
*N*	20	18
Gestational age (weeks)	37.1 ± 2.5	38.2 ± 1.5
Birthweight (g)	2604 ± 753	3090 ± 461
Premature		
No	12(60.0)	15(83.3)
Yes	8(40.0)	3(16.7)
Underlying genetic disorder		
No	10(71.4)	
Yes	4(28.6)	
Prenatal diagnosis		
No	11(55.0)	
Yes	9(45.0)	
Obstetric FU		
No	1(5.0)	
Yes	19(95.0)	
Gender N (%)		
Male	13(65)	8(44.4)
Female	7(35)	10(55.6)
Type of conception N (%)		
Normal	18(90)	16(88.9)
IVF	2(10)	2(11.1)
Bethesda classification		
Simple	0	
Moderate complexity	13(65.0)	
Complex	7(35.0)	
Type of delivery		
Vaginal	11(55)	11(61.1)
Cesarean	9(45)	7(38.9)
Liver pathology		
No	16(80)	
Yes	4(20)	
SGA		
No	13(65)	17(94.4)
Yes	7(35)	1(5.6)
Prostaglandin-dependent CHD		
No	6(30)	
Yes	14(70)	
Inotrope administration		
No	4(20)	
Yes	16(80)	
Hemorrhage		
No	17(85)	
Yes	3(15)	
Thrombosis		
No	18(90)	
Yes	2(10)	
Outcome		
Survival	11(73.3)	
Non-survival	4(26.7)	

The values are presented as the mean and standard deviation or number of patients and percentage in brackets. Abbreviations: FU—follow-up; IVF—in vitro fertilization; SGA—small for gestational age.

**Table 2:** Patient diagnoses in the CHD group.

Diagnosis	No
PA/CAVC	1
CoA	4
CoA/VSD	2
CoA/APW	1
TAPVR	1
Ebstein anomaly	1
VSD	1
CCTGA/VSD/PS	1
TGA	1
PA	1
PA/VSD	1
Fallot tetralogy	2
CAVC	1
Single ventricle physiology	1
DORV/VSD	1

Abbreviations: PA—pulmonary atresia; CAVC—common atrioventricular canal defect; CoA—coarctation of aorta; VSD—ventricular septal defect; APW—aortopulmonary window; TAPVR—total anomalous pulmonary venous return; CCTGA—congenitally corrected transposition of great arteries; PS—pulmonary stenosis; TGA—transposition of great arteries; PA—pulmonary atresia; DORV—double-outlet right ventricle.

**Table 3:** Comparison between CHD and control group.

Variable	CHD	Control	*p* Value
Chronological neonatal age (days)	13.8 ± 14.0	4.6 ± 2.2	-
PT (s)	14.4 ± 3.8	11.9 ± 1.8	**0.012**
aPPT (s)	29.7 ± 3.2	29.5 ± 2.7	0.809
Fibrinogen (mg%)	258.9 ± 85.2	268.8 ± 55.8	0.677
FII (%)	60.0 ± 16.5	59.8 ± 17.1	0.976
FVII (%)	41.6 ± 22.4	60.1 ± 16.0	**0.006**
FVIII (%)	156.3 ± 47.7	123.7 ± 51.0	**0.049**
FIX (%)	58.7 ± 14.2	61.3 ± 25.0	0.688
FX (%)	56.8 ± 12.9	53.3 ± 12.8	0.419
VWFAg(%)	207.6 ± 73.8	147.8 ± 26.0	**0.027**
Rcof(%)	175.6 ± 64.9	137.1 ± 23.6	**0.022**
Antithrombin (%)	65.0 ± 18.9	63.1 ± 13.3	0.718
Protein C (%)	39.6 ± 14.1	40.1 ± 8.3	0.884
Protein S (%)	53.1 ± 18.2	44.5 ± 11.2	0.091
D-dimers (μg/mL)	1.8 ± 1.6	6.4 ± 7.5	**0.036**
ADAMTS-13 (ng/mL)	490.4 ± 167.4	577.2 ± 113.6	0.073

Data are expressed as mean values and standard deviation. *p* < 0.05 was considered statistically significant. Values presented in bold are statistically significant. Abbreviations: PT—prothrombin time; APTT–activated partial thromboplastin time; VWF Ag—von Willebrand factor antigen; Rcof—ristocetin cofactor activity.

**Table 4:** Comparison between non-prostaglandin-dependent and prostaglandin-dependent CHD.

Variable	Non-Prostaglandin Dependent	Prostaglandin Dependent	*p*-Value
PT (s)	11.4 ± 0.5	16.0 ± 4.0	**0.022**
FVII (%)	68.6 ± 11.6	29.2 ± 15.8	**<0.001**
FX (%)	72.0 ± 12.2	51.0 ± 8.5	**0.002**
Protein C (%)	50.6 ± 7.9	34.1 ± 12.8	**0.017**
Protein S (%)	67.8 ± 11.3	47.1 ± 18.0	**0.031**
ADAMTS-13 (ng/mL)	503 ± 127	452 ± 126	0.455
D-dimers (μg/mL)	1.0 ± 0.7	2.2 ± 1.8	0.181

Data are expressed as mean values and standard deviation. *p* < 0.05 was considered statistically significant. Values presented in bold are statistically significant. Abbreviations: PT—prothrombin time.

## Data Availability

The data presented in this study are available on request from the corresponding author. The data are not publicly available due to privacy.
